# The Effects of Force That Pushes Forward Lumbar Region on Sagittal Spinal Alignment When Wearing Backpack

**DOI:** 10.3390/ijerph16193643

**Published:** 2019-09-28

**Authors:** Tae-sung In, Seung-man Yu, Sang-hun Jang

**Affiliations:** 1 Department of Physical Therapy, Gimcheon University, 214, Daehak-ro, Gimcheon 39528, Korea; 2 Department of Radiological Science, Jeonju University, 303, Cheonjam-ro, Wansan-gu, Jeonju-si 55069, Korea

**Keywords:** spine, lumbar, alignment, backpack

## Abstract

The purpose of this study is to design a backpack to push the lumbar region forward and confirm the change in the sagittal plane of the spine using radiography when wearing the backpack to present an effective backpack wearing method that can help spinal alignment. Place the question addressed in a broad context and highlight the purpose of the study. A total of 14 adult volunteers participated in the study. The study was carried out on the subjects without carrying a backpack, with a general backpack, and with a backpack designed to push the lumbar region forward. We investigated cervical, thoracic, lumbar, and sacral alignment under these three conditions. Lumbar lordosis showed a significant decrease in the state of wearing a general backpack compared to the case without a backpack, and a significant increase in the state of wearing a backpack designed to push the lumbar region forward rather than a general backpack. In addition, the sacral slope was significantly increased when carrying the backpack designed to push the lumbar region forward, compared to carrying the general backpack. There was a significant correlation between the sacral and lumbar alignment change when wearing the backpack compared to the state without a backpack. The results of this study indicate that wearing a backpack designed to push the lumbar region forward may contribute to the recovery of lumbar lordosis that is reduced when wearing a general backpack. This may be due to an increase in the sacral slope corresponding to the inferior angle of lumbar spine.

## 1. Introduction

Backpack is a piece of commonly used carrying equipment, and many studies have recently reported that it may adversely affect the spine [1]. In addition, the number of patients with spinal alignment deformity has been increasing recently [2]. Adolescents and adults have different major problems in spinal deformity, so treatment is also different. In adolescence, deformation correction is the typically the main purpose of the treatment, whereas pain relief is the first priority for the treatment of adult spinal deformity [2].

Spinal deformity does not occur in the sagittal plane or frontal plane alone, but rather in a complex form. Most of the major causes of spinal diseases are closely related to the shape of the sagittal plane as well as the alignment of the frontal plane [3]. Much research has been carried out with methods such as the Cobb angle measurement in the frontal plane, and the understanding of lumbar alignment has progressed accordingly. There are also many studies on the pathology and treatment of normal alignment and pathological curvature [4]. However, the research on the sagittal plane is relatively insufficient compared to the frontal plane, and the mechanism of abnormal curvature has not been investigated much.

There are many factors related to sagittal plane alignment of the spine when the posture deformity is examined. A previous study reports that as the Internet usage rate increased, people spend a lot of time in front of the computer, which caused lumbar lordosis to disappear, and the restoration of lumbar lordosis is important in the restoration of the balance in the sagittal plane of the spine [5], and it is also reported that the decrease in lumbar lordosis was associated with poor health-related quality of life [6]. When the monitor is positioned lower than eye level, the head moves forward. The lower cervical region is bent forward and the upper cervical region is bent backwards. This is called forward head posture or turtle neck syndrome [7,8], and this causes dysfunction of the musculoskeletal system in the neck and shoulders [9,10]. It is also reported that such a posture can put a strain on the neck, cause myofascial pain syndrome, and also may cause headaches [11]. 

Backpacks commonly used by students and adults have also been reported to contribute to the increase in lumbar and cervical deformity mentioned above. As a result, it has been reported that adults feel a sense of heaviness, fatigue, and even lower back pain due to backpacks [12]. The backpack reduces the immediate change in sagittal spinal curvature in upright stance, especially the lordosis of the lumbar spine and causes a decrease in normal repositioning ability. This decrease contributes to an increased risk of spinal injury even after the backpack is removed. [1]. Based on the above studies, the health of the spine is closely related to the backpack. and in this regard, it is very important to suggest an effective way of wearing the backpack to maintain balance in the sagittal plane of the spine.

The purpose of this study is to design a backpack to restore the decrease in lumbar lordosis which negatively affects the spinal balance and confirm the change in the sagittal plane of the spine using radiography to present an effective backpack wearing method that can be beneficial to spinal alignment. 

## 2. Materials and Methods

### 2.1. Subjects

A total of 14 adult volunteers participated in the study. Subjects were between 23 and 28 years of age and were fully informed about the study and gave their consent to participate in the study. This study was approved by Gimcheon University Bioethics Committee (GU-201709-HRa-06-02). Subjects were excluded from the study if they had a medical history of spine such as chronic back pain, deformity, spondylolysis, spondylolisthesis, scoliosis, and Scheuermann’s kyphosis.

### 2.2. Protocol

The subjects participated in the study without carrying a backpack, carrying a general backpack, and carrying a backpack designed to push the lumbar region forward. The backpack is a general two strap backpack, 300 mm wide, 450 mm tall, and 100 mm in length front and back, with a Velcro attached to the back of the backpack, where the Styrofoam designed in this study can be attached and detached. The Styrofoam is 300 mm wide, 120 mm long, and 36 mm high, with rounded parts that contact the back to smoothly apply the force pushing the lumbar region forward. If Styrofoam is not attached, it is a general backpack, and if a Styrofoam is attached, it is a backpack designed to push the lumbar region forward. The highest part of the Styrofoam reached the L2 site, and was attached to the Velcro of the back plate.

In the previous study, the weight of a backpack was mainly studied in the range of 5%, 10%, and 15% of the weight of the subjects, and it was found that the weight of a backpack more than 10% of the subject’s weight affected the subject’s spine [13]. Therefore, in this study, the total weight of backpack was determined to be 10% of the subject’s weight. In order to adjust the weight of the backpack at 10% of the subject’s weight, an acrylic plate (280 m by 280 m by 10 m), which is a human body equivalent material, was used in the backpack. The weight of the backpack was adjusted with the increase and decrease in the number of acrylic plates. A large area of human body equivalent material, acrylic phantom, was used to prevent the overall weight of the backpack from leaning toward a specific direction.

Radiographic images were obtained in all three states, without carrying the backpack, with the general backpack, and with the backpack designed to push the lumbar region forward. In order to acquire the lateral projection radiograph of the whole spine of the subject, the teleradiograph method was used by digital X-ray radiography (ysio-max, Siemens, German). For the test method, the centerline of the X-ray is positioned between thoracic spine seven–10 according to the subject’s height, and the two images of X-ray in upper and lower directions were stitched to express the whole image of the spine. To minimize distortion of the image caused by X-ray diverging, the focus to detector distance (FID) was set to 90 inches and the external auricular meatus and femur heads were used as reference points in the x-ray field size to include the whole spine of the subject. In order for the weight of the backpack used in this study to be evenly distributed throughout the spine, the subject should stand with his feet spread shoulder-width wide, and the subject was instructed to perform weight-bearing, keeping the knees unbent. In order to minimize the overlap of the shoulder and humerus with the thoracic spine column in the image, the angle between the humerus long axis and the coronal plane forms about 30 degrees by placing the subject’s hand on the support positioned at the shoulder height.

### 2.3. Measurement

Spinal alignment indicators were measured and analyzed using 3D radiography measurement software (Xelis, Infinity, Seoul, Korea) based on radiographic images taken in three conditions: Without a backpack, with wearing a general backpack, and with wearing a backpack designed to push the lumbar region forward.

Experienced radiologists with more than two years of clinical experience measured cervical, thoracic, lumbar, and sacral alignment. In order to examine the cervical alignment, the lower cervical lordosis, that is, the angle formed by the two tangent lines drawn at the C2 posterior corner and C7 posterior corner were measured. Since thoracic kyphosis angle is less visible because the one–four thoracic spine is hidden from the shoulder, the angle between the fifth thoracic vertebrae superior border and the 12th thoracic vertebrae inferior border was measured. The lumbar lordosis angle was measured between the first lumbar vertebrae superior border and the first sacral vertebrae superior border. The sacral slope angle is also the angle formed between the superior endplate of first sacrum and the horizontal line [14].

### 2.4. Statistical Methods

Statistical analysis of all collected data was done using the SPSS version 20.0 statistics program for Windows (IBM Corp., Armork, NY, USA). The general characteristics of the subjects were presented as mean and standard deviation of all variables measured using descriptive statistics. Repeated measures ANOVA was used to compare each spinal alignment in three states. In addition, in order to investigate the correlation between the spinal alignment without wearing a backpack and with wearing a general backpack, and also the correlation between the alignment without wearing a backpack and with wearing a backpack designed to push the lumbar region forward, the bivariate coefficient of correlation was calculated. For the test of the statistical significance, the significance level α was set to 0.05 for all analyses.

## 3. Results

### 3.1. General Characteristics of Subjects

The subjects were 14 male adults with 24.43 ± 1.50 of age, and their height was 173.64 ± 5.69 cm, and their weight was 73.44 ± 7.92 kg.

### 3.2. Comparison of Cervical Alignment

Radiographs were analyzed to compare the lower cervical alignment in all three conditions: Without the backpack, the general backpack, and the backpack designed to push the lumbar region forward. Repeated measures ANOVA results showed no significant differences between all three conditions (Table 1).

### 3.3. Comparison of Thoracic Alignment

Thoracic kyphosis was measured in the three conditions, and repeated measures ANOVA results showed no significant differences between them (Table 2).

### 3.4. Comparison of Lumbar Alignment

Lumbar lordosis was measured in three conditions and repeated measures ANOVA results showed statistically significant differences. There was a significant decrease in the state of wearing a general backpack compared to the state without carrying a backpack, and a significant increase in the state of wearing a backpack designed to push the lumbar region forward compared to the state of wearing a general backpack (Table 3).

### 3.5. Comparison of Sacral Alignmentt

The sacral slope was measured in the three conditions and the repeated measures ANOVA results showed statistically significant differences. The sacral slope increased significantly with the backpack designed to push the lumbar region forward, rather than the case of carrying a general backpack and without a backpack (Table 4).

### 3.6. Correlation of Spinal Alignment Changes with and without Carrying a Backpack

In this study, the analysis of correlation of the spinal alignment changes between the condition without a backpack and the condition with carrying a general backpack showed a significant correlation in sacral alignment change and lumbar alignment change. Also, the analysis of correlation of the spinal alignment changes between the condition without a backpack and the condition with carrying a backpack designed to push the lumbar region forward showed a significant correlation in sacral alignment change and lumbar alignment change (Table 5).

## 4. Discussion

Recently, the number of patients with spinal alignment deformity is increasing, and it is known that a backpack, the article-carrying equipment commonly used by adults, contributes to the spinal alignment deformity. In particular, it reduces the normal curve of lumbar, which also affects the overall spinal alignment [1]. Therefore, in this study, we designed a backpack that can increase the lumbar curve of the backpack, especially lumbar lordosis and present an effective backpack wearing method through which can restore the balance in the sagittal plane of the spine.

Previous studies have used nonradiating methods to examine spinal alignment. To measure the frontal spinal curve, a scoliometer was used and for the lateral curves, that is, for the measurement of thoracic kyphosis and lumbar lordosis, kyphometer was utilized [15], or to investigate the spinal alignment, a photogrammetric method was used for the measurement of scoliosis [13]. However, these previous studies had limitations in that for the measurement of sagittal and frontal curvature, a simple back surface was utilized rather than properly using radiographs. It was reported that this limitation led to the difficulties in accurately examine the spinal alignment and also clinical features such as pain [15]. In particular, research using radiographic images is rare under the condition of subjects wearing a backpack. However, a radiological method is needed to accurately analyze spinal alignment, and recently, a method of safely applying it to healthy adults has been proposed [14]. For this study, radiographs were used to more accurately investigate changes in the sagittal plane of the spine after wearing a backpack, and a backpack wearing method that can help the sagittal plane alignment of the spine was presented.

In a previous study, the experiment was conducted with the weight of a backpack at 10% of body weight of the subject to determine the effect of backpack on spinal alignment [13]. The weight of the backpack had a significant effect on spinal pain, and affects posture and gait behavior [13,16]. As a result of analyzing the standing posture and gait pattern with carrying a backpack, it was observed that thorax flexion was increased, the activity of erector spinae decreased, and the activity of abdominals increased and they recommended that the backpack weight should not exceed 10% of the subject’s body weight in terms of spinal alignment and muscle activity [16]. Therefore, also in this study, the minimum backpack weight to observe physical changes was set to 10% of the body weight. It was reported that dorsal pain increased as the weight of the backpack increased, and the method of whether to carry the backpack on one side or on both sides was independent of dorsal pain and low back pain [15], and also in this study, spinal alignment was measured with wearing the backpack on both sides, which is a common way of wearing a backpack.

For the measurement of lumbar lordosis, the region between the first lumbar spine superior border and the first sacral spine superior border was measured. In a previous study, a mean lumbar lordosis of a typical Korean was known to be about 49 degrees [17], and this study also showed a similar angle to the previous study result. In this study, lumbar lordosis was decreased in the state of wearing a general backpack compared to the state of not wearing a backpack and it was increased in the state of wearing a backpack designed to push the lumbar region forward compared to the state of wearing a general backpack. Previous studies have reported that wearing a backpack reduces lumbar lordosis because the body tilts forward while flexing lumbar to move the load behind the back into the base of support (BOS) and this study also showed similar results in terms of wearing a backpack decreasing a normal lumbar lordosis [1]. As the lumbar lordosis decreases, it adversely affects the overall health of the spine [6]. Lumbar lordosis restoration can be said to be very important in the restoration of the sagittal plane balance of the spine [5]. Also, in this study, lumbar lordosis was increased when the backpack designed to push the lumbar region forward was worn compared to the case of a general backpack, and this indicates that wearing the specially designed backpack was effective in restoring the lumbar lordosis which can be decreased by wearing a general backpack.

The sacral slope angle showed a significant increase in the state of wearing the backpack designed to push the lumbar region forward than wearing the general backpack. This means that the backpack designed to push the lumbar region forward contributed to the restoration of lumbar lordosis, but this was mainly done at the L5-S1 level. Sacral slope can be thought of as the inferior angle of the lumbar spine because the lower arc of lumbar lordosis is geometrically identical to the sacral slope [3]. As we go down the lumbar spine, the greater the angle of lordosis [18], and in the case of this study, a large part of lumbar lordosis also corresponded to the sacral slope. If the lordosis of the upper lumbar spine is reduced due to improper posture, it is known that the lumbar lordosis of the lower lumbar is overextended as a compensatory action and the total lumbar lordosis is increased to some extent to restore the sagittal plane balance [19]. According to the results of this study, carrying a backpack reduces lumbar lordosis, but the backpack designed in this study is thought to have played the above role. Previous studies have reported that the cases of either too large or too small sacral slopes can cause problems. For example, if the slope was between 35 degrees to 45 degrees, symptoms rarely occur, but if the sacral slope is smaller than 35 degrees, disc herniation symptoms can occur, and if the slope is larger than 45 degrees, spinal stenosis may occur [3]. The backpack designed in this study was considered to have produced excessively lordosis in the lower lumbar spine in the light of previous studies, which may cause spinal stenosis when the posture is maintained for a prolonged time [3]. Therefore, it is thought to be appropriate to apply the force in the direction of restoring the upper lumbar lordosis rather than focusing only on the restoration of lumbar lordosis in the lower lumbar spine.

Thoracic kyphosis is measured between the superior border of the fifth thoracic vertebrae and the inferior border of the 12th thoracic vertebrae, and because the one–four thoracic spine is hidden from the shoulder, radiographic images are based on the fifth thoracic vertebrae. In general, the normal range of thoracic kyphosis is known to be 20–40 degrees. In the results of this study, the kyphosis angle was measured within the range indicated in the previous study. Previous studies have reported that cervical lordosis and lumbar lordosis affected thoracic kyphosis for balance [14]. There are also some previous studies reporting significant differences in thoracic kyphosis depending on whether the subject was carrying a backpack or not [20]. However, there was no significant difference in this study.

Wearing a backpack tends to increase head-on-neck extension, that is, upper cervical lordosis. This is because wearing a backpack causes lumbar flexion and the tilting of the body forward to move the center of gravity (COG) into the BOS, and upper cervical lordosis increases in order to keep the eyes’ view of the subject forward [1]. Upper cervical lordosis and lower cervical lordosis have negative correlations with each other [21]. Therefore, lower cervical lordosis is reduced when wearing a backpack. In this study, however, lower cervical lordosis did not show statistically significant difference after wearing the backpack. It is thought that this result is different from previous studies because the measurement was carried out with the shoulder joint flexed. Lower cervical lordosis is known to decrease as the head position moves further forward [22], and in the future, if we devise a way to increase lower cervical lordosis after wearing a backpack, it may have a positive effect on improving the overall health of the neck and shoulder.

In this study, as a result of analyzing the correlation of spinal alignment change between the state without a backpack and the state with wearing a general backpack, there was a significant correlation between sacral alignment change and lumbar alignment change. Also, as a result of analyzing the correlation of spinal alignment change between the state without a backpack and the state with wearing a backpack designed push the lumbar region forward, there was also a significant correlation between sacral alignment change and lumbar alignment change. In a previous study, sacral slope and lumbar lordosis were reported to be closely associated [23]. The results of this study also indicate that the sacral slope played a significant role in changing the lumbar alignment when wearing a backpack.

The balance of pelvic and lumbar region has a strong correlation with pain, dysfunction, deformity and degenerative diseases [24,25], and cervical spine sagittal balance has been reported to have a correlation with cervical diseases such as cervical degenerative disc diseases and spondylotic myelopathy [26,27]. However, it is important to simultaneously analyze muscle activity as well as spinal alignment in order to understand these conditions accurately. In this study, the spinal alignment was accurately analyzed by radiological method, but, due to the technical difficulties in removing the noise caused by the electromyography (EMG) electrode contact with the backpack, there is a limitation in not being able to investigate the muscle activity around the spine. In order to find out the overall condition of the spine, not only the alignment status but also muscle activity is an important factor. Therefore, it is necessary to carry out multifaceted research that can investigate not only spinal alignment but also muscle activity while wearing a backpack after making an improvement on the above technical limitations.

## 5. Conclusions

In conclusion, the results of this study suggest that wearing a backpack designed to push the lumbar region forward may contribute to the restoration of lumbar lordosis which is reduced when a general backpack is worn. It is thought that this change in lumbar alignment may be due to the change in the sacral slope.

## Figures and Tables

**Table 1:** Comparison of cervical alignment according to backpack carriage and type.

Parameter	WBC	GBC	PBC	F	*p*
Lordosis (degree)	14.48 ± 10.51	17.83 ± 9.12	18.76 ± 10.62	1.191	0.320

Values are expressed as mean ± standard deviation. WBC, without backpack condition; GBC, general backpack condition; PBC, backpack designed to push the lumbar region forward.

**Table 2:** Comparison of thoracic alignment according to backpack carriage and type.

Parameter	WBC	GBC	PBC	F	*p*
Kyphosis (degree)	27.77 ± 8.49	29.78 ± 7.64	27.96 ± 8.40	0.759	0.478

Values are expressed as mean ± standard deviation. WBC, without backpack condition; GBC, general backpack condition; PBC, backpack designed to push the lumbar region forward.

**Table 3:** Comparison of lumbar alignment according to backpack carriage and type.

Parameter	WBC	GBC	PBC	F	*p*
Lordosis (degree)	46.30 ± 12.45 ^#^	43.03 ± 12.39 *^†^	48.29 ± 11.61 ^#^	9.783	0.001

Values are expressed as mean ± standard deviation. WBC, without backpack condition; GBC, general backpack condition; PBC, backpack designed to push the lumbar region forward. * indicates a significant difference compared with WBC, ^#^ indicates a significance difference compared with GBC, ^†^ indicates a significance difference compared with PBC.

**Table 4:** Comparison of sacral alignment according to backpack carriage and type.

Parameter	WBC	GBC	PBC	F	*p*
Slope (degree)	33.52 ± 12.52 ^†^	34.54 ± 12.20 ^†^	37.35 ± 9.65 *^#^	7.862	0.002

Values are expressed as mean ± standard deviation. WBC, without backpack condition; GBC, general backpack condition; PBC, backpack designed to push the lumbar region forward. * indicates a significant difference compared with WBC, ^#^ indicates a significance difference compared with GBC, ^†^ indicates a significance difference compared with PBC.

**Table 5:** Correlation of spinal alignment changes with and without carrying a backpack.

	GBC-WBC				PBC-WBC			
	1	2	3	4	1	2	3	4
Cervical (1)		0.376	−0.381	−0.496		−0.160	0.107	0.312
Thoracic (2)			−0.470	−0.434			−0.295	−0.019
Lumbar (3)				0.743 *				0.749 *
Sacral (4)								

WBC, without backpack condition; GBC, general backpack condition; PBC, backpack designed to push the lumbar region forward. * Significant with *p* < 0.05 by Pearson test.
